# Mesenchymal Stem Cell Application and Its Therapeutic Mechanisms in Intracerebral Hemorrhage

**DOI:** 10.3389/fncel.2022.898497

**Published:** 2022-06-13

**Authors:** Guoqiang Yang, Xuehui Fan, Maryam Mazhar, Sijin Yang, Houping Xu, Nathupakorn Dechsupa, Li Wang

**Affiliations:** ^1^Research Center for Integrated Chinese and Western Medicine, The Affiliated Traditional Chinese Medicine Hospital of Southwest Medical University, Luzhou, China; ^2^Molecular Imaging and Therapy Research Unit, Department of Radiologic Technology, Faculty of Associated Medical Sciences, Chiang Mai University, Chiang Mai, Thailand; ^3^Department of Acupuncture and Rehabilitation, The Affiliated Traditional Chinese Medicine Hospital of Southwest Medical University, Luzhou, China; ^4^Key Laboratory of Medical Electrophysiology, Ministry of Education and Medical Electrophysiological Key Laboratory of Sichuan Province, Collaborative Innovation Center for Prevention of Cardiovascular Diseases, Institute of Cardiovascular Research, Southwest Medical University, Luzhou, China; ^5^First Department of Medicine, Medical Faculty Mannheim, University Medical Centre Mannheim (UMM), University of Heidelberg, Mannheim, Germany; ^6^National Traditional Chinese Medicine Clinical Research Base and Drug Research Center of the Affiliated Traditional Chinese Medicine Hospital of Southwest Medical University, Luzhou, China; ^7^Institute of Integrated Chinese and Western Medicine, Southwest Medical University, Luzhou, China; ^8^Preventive Treatment Center, The Affiliated Traditional Chinese Medicine Hospital of Southwest Medical University, Luzhou, China

**Keywords:** intracerebral hemorrhage, mesenchymal stem cells, brain injury, neuroprotection, immunomodulators

## Abstract

Intracerebral hemorrhage (ICH), a common lethal subtype of stroke accounting for nearly 10–15% of the total stroke disease and affecting two million people worldwide, has a high mortality and disability rate and, thus, a major socioeconomic burden. However, there is no effective treatment available currently. The role of mesenchymal stem cells (MSCs) in regenerative medicine is well known owing to the simplicity of acquisition from various sources, low immunogenicity, adaptation to the autogenic and allogeneic systems, immunomodulation, self-recovery by secreting extracellular vesicles (EVs), regenerative repair, and antioxidative stress. MSC therapy provides an increasingly attractive therapeutic approach for ICH. Recently, the functions of MSCs such as neuroprotection, anti-inflammation, and improvement in synaptic plasticity have been widely researched in human and rodent models of ICH. MSC transplantation has been proven to improve ICH-induced injury, including the damage of nerve cells and oligodendrocytes, the activation of microglia and astrocytes, and the destruction of blood vessels. The improvement and recovery of neurological functions in rodent ICH models were demonstrated *via* the mechanisms such as neurogenesis, angiogenesis, anti-inflammation, anti-apoptosis, and synaptic plasticity. Here, we discuss the pathological mechanisms following ICH and the therapeutic mechanisms of MSC-based therapy to unravel new cues for future therapeutic strategies. Furthermore, some potential strategies for enhancing the therapeutic function of MSC transplantation have also been suggested.

## Introduction

Intracerebral hemorrhage (ICH) comprises 10–15% of all strokes; more than two million patients worldwide per year suffer from this hemorrhagic type of stroke with a complex pathophysiology and 1-month mortality of about 70% (Keep et al., 2012; Biffi et al., 2015; Bosche et al., 2020; Schrag and Kirshner, 2020; Jain et al., 2021; Rajashekar and Liang, 2021; Witsch et al., 2021). With the development of medical knowledge, the ICH incidence showed an increasing trend worldwide (van Asch et al., 2010; Wu and Anderson, 2020), resulting from the spiraling increment of older adults; the application of anticoagulants, antiplatelets, and thrombolytics; and other issues (Carpenter et al., 2016; Wu et al., 2019). Although significant progress in potential treatment after ICH models has been developed in preclinical research (Bentz et al., 2010; Xiong et al., 2014; Alharbi et al., 2016; Chen-Roetling et al., 2021), the lack of available evidence-based therapeutic strategies still limits the improvement of ICH prognosis in the clinical setting, where only active first-stage rehabilitation and general rehabilitation may lead to a modification of the outcomes (Saulle and Schambra, 2016).

Stem cell therapy is a promising method that has been actively applied in preclinical research of neurological diseases in recent years. Mesenchymal stem cells (MSCs) possess unique properties, including extensive proliferation and differentiation potential, simplicity of acquisition from various sources, low immunogenicity, secretome for extracellular vesicles (EVs), immunomodulation, and anti-inflammatory properties (Schipani and Kronenberg, 2008; Zheng et al., 2018; Dornen and Dittmar, 2021). Until now, MSC therapy has been recognized as a promising therapy in regenerative medicine research and tissue engineering (Bentz et al., 2010; Zheng et al., 2018).

Previously, accumulated evidence from preclinical studies has confirmed the protective effects of MSC therapy after ICH. However, the exact mechanisms of MSC transplantation in clinical translation are still undefined. Therefore, here, we have summarized the mechanisms of MSC application that facilitate neurological restoration after ICH. Simultaneously, some current challenges such as ICH-induced mass effect, iron overload, inflammation, oxidative stress, and limitations of clinical translation for MSC therapies are also emphasized when MSC treatment is applied to the ICH.

## Pathological Changes After Intracerebral Hemorrhage

The brain injury after ICH, which always manifests a high risk of ischemia and recurrent bleeding (Baang and Sheth, 2021), is traditionally described as a cascade of disease courses including two different successive pathological processes: the primary brain injury stage and the secondary brain injury stage (Huang et al., 2020). In the first few hours of the primary brain injury stage after ICH occurs, the blood released from the ruptured blood vessels develops to form a consistent mass effect. Hematoma formation induces mechanical dissection and compression of the brain tissue, leading to high intracranial pressure forming herniation (Huang et al., 2020), which is always addressed by advanced surgical techniques in clinical practice (Tschoe et al., 2020). The secondary brain injury (SBI) stage is composed of damage caused by hemolytic products of erythrolysis, excitotoxicity, oxidative stress, and neuroinflammation-induced neurological deficits (Aronowski and Zhao, 2011; Babu et al., 2012; Duan et al., 2016; Lan et al., 2017), accompanied by successive pathological changes, including hemodynamic change-induced ischemia, enhancement of cerebral edema, destruction of the blood–brain barrier (BBB), and direct cellular toxicity (Aronowski and Zhao, 2011; Keep et al., 2012; Xiong et al., 2014; Chen S. et al., 2015; Mohammed Thangameeran et al., 2020; Zheng et al., 2022). During this process, the hemoglobin, heme, and free iron, released from erythrolysis and other blood derivatives, infiltrate into the perihematoma, activating microglia/macrophages to accelerate hematoma clearance displaying neuroprotection (Bosche et al., 2020; Lua et al., 2021; Wei et al., 2022; Zheng et al., 2022). Thrombin activation after ICH implies the vasculogenic edema formation associated with the destruction of endothelial cells and the BBB (Wilkinson et al., 2018). Perihematomal edema (PHE), associated with a worse prognosis, has been recognized as an evident marker of SBI after ICH and a likely therapeutic pathophysiological target for attenuating SBI (Brouwers and Greenberg, 2013; Bautista et al., 2021; Chen et al., 2021). Furthermore, it was displayed that intracranial hematoma expands to further and adjacent brain tissues through perivascular spaces, white matter tracts, and their perineurium (Yin et al., 2013; Fu et al., 2021), particularly if ICH is combined with an intraventricular hemorrhage (Bosche et al., 2020). All these pathological changes in the hematoma site make the nerve fibers distended and distorted and finally disrupted to a point at which they cannot be rescued. Therefore, the pathological changes of SBI after ICH induced permanent impairment of brain tissues, and severe neurological deficits should continuously be paid close attention to Qureshi et al. (2009) and Wilkinson et al. (2018).

**FIGURE 1:**
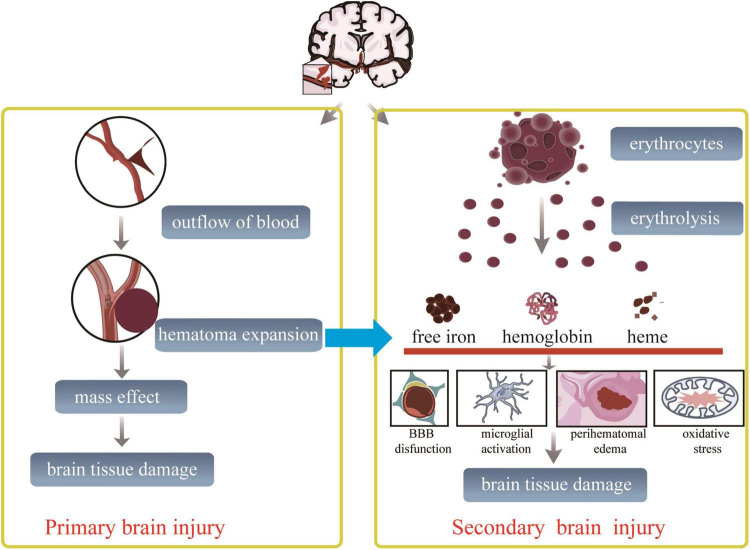
Proposed schematic diagram linking outcomes after ICH. After ischemic cerebral hemorrhage onset, primary and secondary brain injury is going. The prior brain injury mechanism starts from an occulted blood vessel. And is followed by blood vessel ruptured, extravasated red blood cells causing dynamic hematoma expansion, resulting in the adjacent brain tissue immediately compressed, followed by brain tissue damaged finally. This process disrupts the surrounding brain structures, resulting in early neurological dysfunction. The multiple hemolytic products, including ferrous ions, hemoglobin, heme, and other lytic molecules, cause secondary brain injury within the period from hours to days after the primary brain injury. During this process, nerve and glial cells suffer from oxidative stress, inflammation, excitotoxicity, and death signals.

The pathophysiological changes after ICH are mainly characterized by demyelination and axonal injury. Oligodendrocytes are the only source of myelin formation, insulating myelin sheaths for neurons to enhance the propagation of action potentials and protect the integrity of neurons and axons (Bacmeister et al., 2020). After ICH, there is a sharp increase in intracellular Fe^2+^ released from the hemoglobin of dead erythrocytes, which destroy oligodendrocytes. Demyelination and axonal damage are observed at the edge of the hematoma 6 h after ICH that peaks at the highest level of impairment at 3 days, and the axonal damage gradually extends to the adjacent parenchyma over time (Wasserman and Schlichter, 2008; Fu et al., 2021). Zille et al. (2017) have verified that the hemin and hemoglobin-induced toxic mediators from lysed blood after ICH participate in the death of primary cortical neurons through ferroptosis and necroptosis, rather than caspase-dependent apoptosis or autophagy *in vitro* and *in vivo*. Moreover, Palumbo et al. (2021) observed a time-dependent morphological degeneration of axons in hemin-induced primary cortical neurons, resulting in a declination of the axon area and enhancement of axonal swelling and fragment areas depending on a novel microfluidic device and a deep learning tool on microscopy. Hemorrhages in the internal capsule block the axonal transport to induce axonal dysfunction closely associated with an early decline of motor performance in mice with ICH (Hijioka et al., 2016). The capacity of proliferation and differentiation to oligodendrocyte precursors [NG2(+) Olig2(+)] presented a dramatically increasing trend in the perihematomal region over the first week, which provided the valid re-myelination chance on axon tracts in the rat striatum after ICH (Joseph et al., 2016). Ultrastructural features in mice with injured striatum after ICH examined by transmission electron microscopy at 3, 6, and 28 days have demonstrated remarkable axonal demyelination and degeneration, degenerated neurons, abnormal synapses, and infiltrating macrophages engulfing debris of different degenerated cells (Li et al., 2018). In short, severe destruction of myelin and axons can be seen clearly in rodents with ICH, contributing to the loss of motor function. Therefore, viewing the aforementioned accumulating solid evidence on the ICH, it is critical to point toward the primary brain injury and the second brain injury to benefit from controlling the outcomes following brain injury (Figure 1).

## Different Sources of Mesenchymal Stem Cells in Intracerebral Hemorrhage

Some studies have verified that MSCs can be isolated from various adult tissues such as the bone marrow, adipose tissue, synovium, and neonatal tissues, including the umbilical cord (blood), placenta, amniotic fluid, and amniotic membrane, and possess the potential of differentiating into diverse cell lineages and tissues like bone, adipose tissue, cartilage, nerves, and liver, both *in vivo* and *in vitro* (Gao et al., 2018; Sumer et al., 2018; Mathot et al., 2019; Gong et al., 2021; Li J. et al., 2021; Li Q. Y. et al., 2021; Ramesh et al., 2021; Souza et al., 2021; Zhu et al., 2021; Bian et al., 2022). Others have verified that MSC treatment holding promise exerts indirect therapeutic mechanisms such as anti-inflammation (Huang et al., 2019), secretion of growth factors (Tang W. et al., 2021; Ulpiano et al., 2021), and EVs (Bang and Kim, 2022), which are associated with the recovery of damaged tissues. Moreover, caution is generally needed while using these or similar MSC-related therapeutic approaches (Molcanyi et al., 2013, 2014; Levy et al., 2020; Kim D. et al., 2021). The implantation of stem cells was accompanied by a massive invasion of macrophages into transplantation sites, reactivation of astrocytes, and activated microglia following brain injury inflammatory response, in which the survival rate and integration of implanted stem cells are always impeded (Molcanyi et al., 2007).

Bone-derived MSCs (BMSCs) are frequently used for treating brain injuries not only because of the ease of acquisition from the host but also because of infiltrating capability *via* the BBB without disrupting the structure (Kopen et al., 1999; Gao et al., 2018) to differentiate into neurons or neuron-like cells for tissue reparation (Wislet-Gendebien et al., 2005; Nagai et al., 2007; Bae et al., 2011). Various studies have demonstrated that BMSCs could limit neurological deficits and BBB dysfunction in ICH rats (Chen M. et al., 2015; Wang et al., 2015).

Neonatal tissue-derived MSCs, including human umbilical cord MSCs (HUC-MSCs), umbilical cord blood (HUCB-MSCs), placenta MSCs (HP-MSCs), amniotic fluid (HAF-MSCs), and amniotic membrane (HAM-MSCs), are another kind of widely used MSCs, which have been used to treat neurological deficits in animal models and patients with ICH and displayed apparent therapeutic effects (Nan et al., 2005; Chang et al., 2016; Xie et al., 2016).

Adipose-derived MSCs (ADMSCs) are also used for regenerative medicine isolated from adipose tissues, which are easily accessible and abundant (Gimble et al., 2010). Chen et al. (2012) found that the transplantation of rat ADMSCs for treating ICH rats demonstrated the differentiation of neuron-like and astrocyte-like cells around the injured site and improved the expression level of vascular endothelial growth factor (VEGF) for the recovery of neurological function. Yang et al. (2012) employed the injection of ADMSCs generated from a 65-year-old male donor’s fat tissue into the right femoral vein of ICH-induced stroke rats, which suggested that ADMSC transplantation could facilitate functional recovery of the experimental animals.

Dental pulp stem cells (DPSCs) originate from the neural crest and exhibit neuro-ectodermal features having multilineage differentiation potentials, which were first discovered in the pulp tissue (Gronthos et al., 2000; Pierdomenico et al., 2005). They are a subpopulation of dental pulp cells (DPCs) owing to MSC properties, including the similarity of morphology like fibroblast, adherence, surface marker expression, proliferation, and colony-forming behavior (Lan et al., 2019). Given the attractive characteristics of DPSCs such as ease of acquisition, powerful proliferation ability, and long-time cryopreservation with no loss of multi-directional differentiation capacity, DPSC therapy displays an increasing interest in stroke disease in preclinical and clinical research (Song et al., 2017; Sowa et al., 2018; Lan et al., 2019; Nito et al., 2022). In a preclinical study, Nito et al. (2018) have reported that transplantation of human DPSCs *via* the femoral vein ameliorates infarct volume and motor functional deficits in rats following acute cerebral ischemia. Moreover, the clinical evidence shows that the intracranial transplantation of autologous DPSCs is safe and feasible in patients with chronic stroke, and the maximum tolerable dose is also verified in human subjects, which provides guidance for the design of future clinical trials (Nagpal et al., 2016).

## Administration Routes of Mesenchymal Stem Cells in Intracerebral Hemorrhage

In rodents with ICH, three different ways, including intracerebral, intravenous, and intra-arterial injection, for transplantation of MSCs have often been used (Li J. et al., 2021). Stereotactic intracerebral injection of HUC-MSCs transduced with hepatocyte growth factor (HGF) into the left ventricle improved neurological deficits in rats after ICH, which was due to the improvement of demyelination and axonal regeneration (Liu et al., 2010). Allogeneic and syngeneic BMSCs injected intravenously 24 h after stroke in rats ameliorated the neurological deficits without immunologic sensitization by enhancing the reactive oligodendrocytes and astrocytes or axon–glia units to remodel the injured axons and promote effective reparation of white matter (Li et al., 2006; Rosenzweig and Carmichael, 2015). However, the experiments of Fischer et al. (2009) revealed that after intravenous injection of MSCs, the majority of them were trapped inside the lungs *via* flow cytometry to detect labeled cells reaching the arterial circulation and harvested the lungs, heart, spleen, kidney, and liver. Additionally, many clinical trials have also verified the safety and efficient neuroprotective effects of allogeneic and syngeneic HUC-MSCs, BMSCs, and ADMSCs through intra-arterial injection for different brain diseases such as ICH, ischemic stroke, and traumatic brain injury (Li J. et al., 2021). The adverse events or complications, including microembolisms and decreased cerebral blood flow due to cell dose and infusion velocity, were recently reported after intra-arterial cell delivery in rodent models (Cui et al., 2015). These parameters should be considered before preclinical studies in rats and clinical research in patients with stroke. Cui L. L. et al. (2017) found that MSCs restrained in microvessels occasionally formed conspicuous cell aggregations, giving rise to local blood flow interruptions *in vivo*. Overexpressing integrin α4 (ITGA4) *via* lentiviral transfection on MSCs improved transendothelial migration *in vitro* and furthered safety by alleviating cell aggregations and ameliorating the induced cerebral embolism after intracarotid transplantation of MSCs into rats with stroke (Cui L. L. et al., 2017). Moreover, although the number of clinical trials using MSCs for regeneration and immunomodulation for stroke therapy increases, the fact that the proliferative and immunomodulatory functions of MSCs decrease with aging due to the complex ICH environment cannot be ignored (Li et al., 2017; Fafian-Labora et al., 2019). Furthermore, it has been verified that the condition of the transplanted cells impacts the subsequent therapeutic effects. Weise et al. (2014) have demonstrated that the intravenous administration of cryopreserved human umbilical cord blood mononuclear cells (HUCB-MNCs) did not display neurorestorative properties in spontaneously hypertensive rats with stroke, which suggests that translating cord blood therapy into clinical stroke trials requires further knowledge about its precise functions. Moreover, the immunomodulatory activity of cryopreserved MSCs can be reduced after thawing when compared to freshly prepared MSCs (Moll et al., 2016).

## Mechanisms of Mesenchymal Stem Cells in Intracerebral Hemorrhage

Ongoing research efforts focus on the potential plasticity and therapeutic applications of MSCs in regenerative medicine. Accompanied by the properties of trans-differentiation into lineages derived from the neuro-ectoderm and migration to the injured sites, MSCs have been suggested to be a promising candidate in regenerative medicine (Chen et al., 2008). It is a fact that MSC therapy has demonstrated its function in improving ICH-induced neuronal defects, neural network reconstruction, and neurological functions *via* anti-inflammation, neurogenesis, angiogenesis, and anti-apoptosis (Zhu et al., 2021).

### Promoting Regenerative Repair and Structural Remodeling

Many secreted trophic molecules as components of their secretome from MSCs transplanted by orthotopic and caudal vein injection facilitated the endogenous reparatory mechanism, which ultimately improved the recovery of neurological function after stroke (Smith and Gavins, 2012; Shichinohe et al., 2015; Karagyaur et al., 2021). MSC transplantation is promising for promoting angiogenesis. It has been found that HA-MSCs upregulated the human placental endothelial cells (hPECs) viability due to its crucial angiogenic potential (Pfeiffer et al., 2019). Furthermore, HP-MSCs significantly increased the viability, migration, and network formation of endothelial cells, exerting angiogenic potential depending on released angiogenic factors in vitro, such as VEGF, angiogenin, IL-6, IL-8, and matrix metalloproteinase (MMP)-1 and 2 (Konig et al., 2015). Moreover, HPMSCs have also been found to promote neovascularization *in vivo* (Kinzer et al., 2014; Tuca et al., 2016; Ertl et al., 2018).

Following administration *via* different routes, they migrate to the damaged tissues or organs, where they might face severe surroundings coupled with death signals because of the disordered formation between the cells and matrix. Preconditioning with various physical, chemical, and biological factors; genetic modification; and optimization of MSC culture conditions are pivotal strategies to facilitate their functions *in vitro* and *in vivo*, which will contribute to improving the efficacy of MSC administration in regenerative medicine (Hu and Li, 2018; Zhao et al., 2019).

Preconditioning HUCB-MSCs with hypoxia remarkably enhances their proliferative capacity and the expression of the neural gene GFAP *in vitro* (Kheirandish et al., 2017). Although hypoxia reduces the cell viability and proliferation of MSCs initially, the following reoxygenation process improves their rehabilitation, and the approach of hypoxia and reoxygenation (H/R) promotes the expression of pro-survival genes and the release of various trophic factors in MSCs (Kim et al., 2015). On account of the advantages of H/R, current studies have focused on optimizing oxygen concentrations to promote the cell activities and therapeutic effects of MSCs. Recently, the potential of electroacupuncture (EA) in promoting neurofunctional recovery through the NT4/5-TrkB-CREB signaling pathway has been identified (Ahn et al., 2016). The combined function of EA and transfected MSCs with modified TrkB gene (TrkB-MSCs) has been investigated in mice with stroke (Ahn et al., 2019). Ahn et al. (2019) found that EA can remarkably enhance the survival and differentiation of grafted TrkB-MSCs to neuronal cells *via* the BDNF/NT4-TrkB-CREB signaling pathway. EA directly upregulated the gene expression of plasticity-related gene 5 (PRG5), which is a critical neurogenesis factor, and also upregulated the protein expression of postsynaptic density 95 (PSD95) and synaptophysin (SYP). Moreover, EA downregulated the expression of neurogenesis inhibitory molecules, including NogoA, lysophosphatidic acid, and RhoA, to improve the proliferation and differentiation of endogenous neural stem cells and synaptic plasticity in stroke rats (Tan et al., 2018; Yang et al., 2022). Deng et al. (2021) have reported that EA promoted differentiation of transplanted MSCs into neuron-like cells and expressions of BDNF and NGF proteins displaying therapeutic efficacy in ICH rats. Furthermore, Zhang et al. (2015) suggested that the combination of direct stereotactic intracerebral injection of HUC-MSCs and minimally invasive hematoma aspiration was better than either therapy alone in reducing neuronal damage and improving neuronal functions. Although some benefits of pre-conditioning MSCs have been reported in animal experiments, the experiments of Mello et al. (2020) presented a warning conclusion that intravenous administration of HUC-MSCs decreased the hematoma volume in moderate collagenase-induced brain hemorrhage in rats but failed to reduce the hematoma volume, and continuous neurological impairments can be observed in animals with a severe ICH.

Brain-derived neurotrophic factor (BDNF), recognized as a nerve growth factor and released from MSCs, has been widely researched in different brain diseases, including stroke, neurodegenerative diseases, and others (Ko et al., 2018; Kim H. J. et al., 2021; Sharma et al., 2021). BMSCs overexpressing glial cell-derived neurotrophic factor (GDNF) and placental growth factor (PlGF) display better neuroprotective effects in the rodent model of ICH and cerebral ischemia (Liu et al., 2006; Yang et al., 2011). It has been proved that overexpressing BDNF of HUCB-MSCs induced their neural differentiation via the TrkB-mediated phosphorylated ERKs and β-catenin in the developing brain (Lim et al., 2011). Moreover, others also found that HUCB-MSCs and their secreted BDNF exert therapeutic effects in intraventricular hemorrhage and ameliorate neuronal loss and neurocognitive deficits via the BDNF-TrkB-CREB signaling pathway (Ko et al., 2018). Zhang et al. (2018) have demonstrated a pronounced downregulation of microRNA-21 (miR-21) in ICH patients’ blood and brain tissue. Therefore, they treated ICH rats with the modified MSCs overexpressing miR-21 and observed improved MSC survival that could be conveyed to neurons depending on influent exosomes derived from MSCs, alleviating the neuronal injury by targeting transient receptor potential melastatin 7 (TRPM7) (Zhang et al., 2018). MiR-126-modified MSCs also alleviated the neuronal apoptosis in collagenase-induced ICH rats’ injured brain tissues (Wang et al., 2020). The expression level of growth-associated protein 43 (GAP-43), which is not only known as a growth cone-specific protein to developing neurons but also recognized as a novel axonal phosphoprotein, which has a synchronous effect on BDNF, was highly upregulated on immature growing axonal terminals along with enhancement of synaptic plasticity, but its downregulation suggested the formation of matured synaptogenesis (Gupta et al., 2009; Morita and Miyata, 2013). Cui J. et al. (2017) have reported that MSCs can enhance the expression level of GAP-43 to ameliorate neurological deficits and improve axonal regeneration via the ERK1/2 and PI3K/Akt signaling pathways in rats with ICH. Taken together, it is suggested that MSC therapy can enhance improvement of the axonal damage and synaptic plasticity to some extent, whereas GAP-43 and BDNF may be taken into account as the potential therapeutic target after ICH for MSC treatment.

The corticospinal tract (CST) is the sole descending fiber bundle responsible for conducting the electrical activity, where the axons from nerve cells immediately contact spinal motor neurons through the synaptic connections, associating with the experienced voluntary movement in people and motor function in animals. Critically, early motor dysfunction after stroke attributes to the destruction of the framework and function of axons due to the hemorrhage of the caudate nucleus disrupting the CST in the internal capsule (Hijioka et al., 2016*;* Jang et al., 2018*;* Chen W. et al., 2019), where the injury lasted for at least 5 weeks, suggesting that the structural integrity of the CST has sustained damage in ICH (Ng et al., 2020). Meanwhile, the longitudinal pathological alterations in the cervical portion of the spinal cord of the CST in mice with ICH were also confirmed by confocal microscopy and transmission electron microscopy (Ng et al., 2020). In short, nerve regeneration after ICH mainly depends on axonal sprouting of existing surviving nerve cells, new forming synapses, synaptic plasticity, and nerve growth factors such as BDNF and GAP-43. Many studies have verified that MSCs can facilitate neurogenesis based on their characteristics of differentiation, secretion, and axonal plasticity, which ameliorate worse outcomes after brain injury. MSCs may also hinder hematoma expansion and decrease acute mortality during the hyperacute course in rats with ICH by improving the endothelial integrity to cerebral vasculature and enhancing the tight junction protein levels, including zona occludens-1 (ZO-1) and occludin (Choi et al., 2018). Pretreatment of HP-MSCs with apocynin, an NADPH oxidase inhibitor, enhanced the endovascular integrity of cerebral vasculature, demonstrating therapeutic efficacy in the ICH acute stage (Min et al., 2018). Meanwhile, the experiments conducted by Wang et al. (2020) also reported that miR-126-modified MSCs diminished the mRNA expression of protease-activated receptor-1 and MMP-9 while enhancing the ZO-1 and claudin-5 expression levels for the repairment of BBB in the ICH rats.

### Anti-inflammatory and Immunomodulatory Properties of Mesenchymal Stem Cells

Except for its role in regenerative medicine, MSC therapy has also been experimentally investigated in other indications, including its immunomodulatory capabilities (Diehl et al., 2017). It has been known that MSCs prevent T-cell response indirectly through the modulation of dendritic cells and directly by suppressing the natural killer cell function of CD8^+^ and CD4^+^ T cells. Specifically, they can well restrain or regulate immune responses in complex interactions of T and B lymphocytes, dendritic, and NK cells (Wang and Zhao, 2009). Their immunosuppressive effects of T lymphocyte proliferation, rather than induction of apoptosis, are not only based on soluble factors including transforming growth factor beta 1 (TGFβ1), hepatocyte growth factor (HGF), and other mediators but also depend on direct interactions of cells (Di Nicola et al., 2002; Krampera et al., 2003; Le Blanc et al., 2003). A preclinical investigation was performed by Azevedo et al. (2020) to observe the potential functions of MSCs on CD4 T cells, and the data suggested that MSCs induced CD4 T cells into Treg-like cells *via* TGF-β and/or programmed death-1 (PD-1)/programmed death ligand 1 (PD-L1) pathways. It has been verified that PD-L1 decreased the infiltration of CD4^+^ T cells to the brain and resulted in upregulation of Th2 and Treg cells but downregulation of Th1 and Th17 cells through the mTOR pathway by *in vitro* and *in vivo* experiments (Han et al., 2017). Moreover, other studies have also proved similar results in the ICH rodent model, a well-defined B10.D2 [H-2(d)) donor to BALB/c (H-2(d)] recipient mice model and experimental autoimmune neuritis (EAN) rat model (Fujiwara et al., 2014; Ding et al., 2016; Han et al., 2017).

There is a well-acknowledged fact that neuroinflammation aggravated the progress of ICH-induced brain damage. Thus, the strategy for regulating the immunoreaction could attenuate ICH-induced brain injury. The remarkable properties of anti-inflammation and immunomodulation make MSC transplantation an appropriate therapeutic candidate for responding against inflammatory diseases like ICH through regulating microglia and neutrophils while enhancing the protective function of anti-inflammatory cytokines and inhibiting the disadvantages of pro-inflammatory cytokines. Kim J. M. et al. (2007) found that ADMSC transplantation for the rats’ ICH model could alleviate the acute inflammation and chronic brain degradation to improve long-term functional recovery.

Recent studies have confirmed that MSCs are effective modifiers maintaining a resting microglial phenotype, preventing microglial activation by downregulating pro-inflammatory cytokines/chemokines and upregulating hypoxia-inducible factor 1-alpha (HIF-1α) and growth factors including VEGF, BDNF, GDNF, stromal-derived factor-1 (SDF-1), and erythropoietin (EPO) following stroke (Wei et al., 2012; Yan et al., 2013). Other studies have shown that hypoxic preconditioning augmented MSC survival and tissue-protective capability, displaying better therapeutic efficacy than the single MSC transplantation by improving the miR-326/PTBP1/PI3K-mediated autophagy and alleviated microglial activation to downregulate IL-1β and TNF-α expression levels and microglial pyroptosis after ICH (Liu et al., 2021a,b). Human ADMSCs improved the neurological deficits in the ICH mice model by suppressing the acute inflammation mediated by the CD11^+^CD45^+^ subpopulation of cells (Kuramoto et al., 2019). In the rat middle cerebral artery occlusion (MCAO) model, microglial activation and their pro-inflammatory phenotype were significantly downregulated by interferon-γ (INF-γ)-activated MSCs, along with the improvement in oligodendrogenesis by the upregulation of neuron-glial antigen 2, a hallmark protein of oligodendrocyte progenitor cells, for promyelination and minimizing the infarct and penumbra (Tobin et al., 2020). The microglial activation is mainly regulated by CX3CR1, and some studies have verified that MSCs are known to polarize M1 macrophages to M2 phenotypes *via* CX3CR1 (Min et al., 2016; Li et al., 2019). Du et al. (2020) found that in the global cerebral ischemia mice, downregulated CX3CR1 remarkably alleviated microglial activation and peripheral inflammatory responses, including the downregulation of IL-6, IL-1β, and TNF-α in the serum, which promoted the differentiation and maturation of mature oligodendrocytes from oligodendrocyte progenitor cells in the striatum, cortex, and hippocampus and thus, attenuated further dysfunction of myelin from ischemia-induced brain injury. Similarly, Hamzei Taj et al. (2018) found that after transplantation of genetically modified IL-13-engineered MSCs (IL-13-MSCs) for knock-in fluorescent protein reporter mice (CX3CR1eGFP/^+^ CCR2RFP/^+^), the brain-resident microglia can be recognized from freshly infiltrated macrophages after stroke. The results suggested that the graft of IL-13-MSCs switched the microglia/macrophages into an alternative activation state (an anti-inflammatory and neuroprotective phenotype) by significantly increasing Arg-1 and decreasing MHC-II expression after 14 days of ischemia (Hamzei Taj et al., 2018). Engineered MSCs have also been applied in malignant glioma tumor models (Sun et al., 2011). The exosomes derived from the miR-146a-5p-rich BMSCs could reduce neuronal apoptosis and inflammation *via* inhibiting microglial M1 polarization by downregulating the expression of IL-1 receptor-associated kinase 1 (IRAK1) and nuclear factor of activated T cells 5 (NFAT5) (Duan et al., 2020). Furthermore, miR-183-5p is also implicated in brain injury and found to be decreased in db/db rat brain tissues after ICH (Ding et al., 2021). It has been verified that EVs derived from BMSCs repressed the inflammatory response through the microRNA-183-5p/PDCD4/NLRP3 pathway (Ding et al., 2021; Nakano and Fujimiya, 2021).

Astrocytes, one of the primary components of glial cells, have also been confirmed to have a reparative function in the injured brain tissue after ICH (Zhang et al., 2006; Chen et al., 2020b), although adequate research is not recorded. After transplantation of MSCs in ICH mice, astrocytes underwent astroglial–mesenchymal phenotype switching and became capable of proliferating and were protected from apoptosis *via* downregulation of p-MST1 and p-YAP (Chen et al., 2020b). Together, the Hippo pathway-mediated favorable impacts of MSCs can be recognized as a unique therapeutic target in ICH injury (Chen et al., 2020b). Experiments performed by Chen et al. (2020a) also found that transplanting BMSCs led to an elevation of glial fibrillary acidic protein (GFAP), a biomarker of astrocytes, and the level of expression and improved astroglial–mesenchymal phenotype switching and anti-apoptotic abilities through the Cx43/Nrf2/HO-1 axis. Interestingly, Donega et al. (2014) reported that MSCs transplanted *via* intranasal administration for the hypoxic–ischemic (HI) neonatal mice model migrating specifically toward the lesion site successfully downregulated the expression level of GFAP while decreasing the formation of glial scars, which is a crucial step for promoting neurogenesis. Chen et al. (2020a) maintained that astrocytes serve as the primary defense system, just like a double-edged sword for responding to ICH injury. Fortunately, the graft of MSCs for ICH-injured mice exerting anti-inflammatory and angiogenic properties significantly alleviated the dysfunction of cognition, movement, and hematoma volume, which corresponded with previous studies (Bedini et al., 2018). Comprehending how to enlarge the advantages and minimize the disadvantages of reactive astrocytes is the critical challenge in improving ICH-induced brain injury. Another factor is IL-33, a member of the IL-1 family having pro-inflammatory and anti-inflammatory properties displaying a double-edged sword function that improves wound healing by enhancing M2 macrophage polarization, collagen accumulation, and angiogenesis in the wound sites (Schmitz et al., 2005; He et al., 2017). It is mainly expressed in microglia, astrocytes, and oligodendrocytes in the central nervous system (CNS), leading to severe pathological changes in mucosal organs (Schmitz et al., 2005). Chen Z. et al. (2019) found that IL-33 attenuated neurological deficits, neuronal degeneration, and secondary brain injury by a favorable regulation of ICH-induced microglial responses, which led to microglial polarization from M1 to M2; thus, IL-33 maybe another novel therapeutic target for ICH intervention.

MSCs display immunomodulatory functions by secreting multifunctional paracrine signaling factors, including cytokines, growth factors, and chemokines, the combined effect of which regulates the cellular immune function (Zhang et al., 2013; Zhao et al., 2013; Liu et al., 2014; Zhou et al., 2019). Transplanted MSCs by intravenous infusion can infiltrate through the BBB and express a protein marker phenotype for neuronal cells (Chen et al., 2001). It has been widely accepted that the functional benefits derived from MSC grafts arise from their improvement of the trophic support and anti-inflammatory effect, thereby controlling the potentially toxic environment (Caplan and Dennis, 2006; Hess and Borlongan, 2008). Some investigations suggested that the bystander mechanism of MSC protective functions depends on some soluble factors, including IL-10, indoleamine 2,3-dioxygenase (IDO), PGE2, TGF-β1, TNF-α, and TNF-α-stimulated gene/protein 6 (TSG-6) (Nemeth et al., 2009). Cell-secreted EVs commonly encapsulate these different kinds of molecules, and according to the dimension and origination, they are called exosomes, microvesicles (MVs), and apoptotic bodies. TSG-6, an anti-inflammatory factor, can restrain neutrophils infiltrating into the inflammatory region, display functions on resident macrophages through interaction with the CD44 receptor, and block the NF-κB signaling pathway (Chen M. et al., 2015). Tang B. et al. (2021) reported that TSG−6 derived from BMSCs moderated reactive astrocytes by downregulating the NF−κB signaling pathway to attenuate BBB dysfunction after ICH. The BMSC transplantation from the jugular vein alleviated the inflammatory response *via* decreasing the pro-inflammatory cytokines and alleviated BBB dysfunction in ICH-bearing rats by releasing TSG-6, downregulating the expression levels of iNOS, MMP-9, and peroxynitrite [ONOO (-)] (Chen M. et al., 2015). Recently, researchers have confirmed in the monkey that the treatment with intravenous infusions of MSC-derived EVs (MSC-EVs) alleviated injury-induced hyperexcitability and regulated the relationship between excitation and inhibition around the injured ventral premotor cortex for reducing pathological changes (Medalla et al., 2020). In addition, MSC-EVs increase the conversion from M1-like pro-inflammatory phenotypes to anti-inflammatory M2 phenotypes to trigger the macrophage polarization by downregulating IL-23 and IL-22 (Li J. et al., 2021).

### Reducing Oxidative Stress

The antioxidative characteristics of MSCs have been verified in an Escherichia coli-induced acute lung injury (ALI) in the mouse model (Shalaby et al., 2014). In the pathophysiological condition of ischemia diseases, including ischemic stroke and heart disease, the PI3k/Akt pathway participating in angiogenesis, oxidative stress, and survival of the MSC graft could be further enhanced by preconditioning of MSCs with pharmacological factors, such as statins (Samakova et al., 2019), which can further their survival capacity and properties of secretome, paracrine, and autocrine secretion (Hu and Li, 2018; Samakova et al., 2019).

**FIGURE 2:**
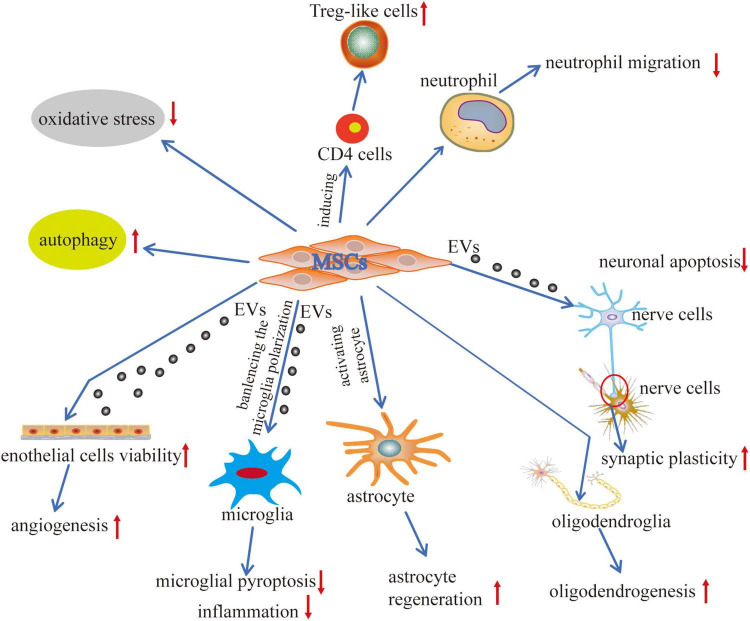
Mechanisms of MSCs application in ICH. The interactions between MSCs and tissue environments have occurred *via* two effective mechanisms cell-to-cell communication and cell-to-extracellular vesicles communication. MSCs interact with the adjacent cells, including the immune cells, nerve cells, glial cells, and endothelial cells, promoting regenerative repair and structural remodeling of damaged tissue. MSCs generate the extracellular vesicles (EVs) that contain lipids, proteins, microRNAs (miR), and cytokines representing an efficient way to transfer functional cargoes between each other. Biological processes are positively modulated, including autophagy, pyroptosis, apoptosis, angiogenesis, inflammation, cell plasticity, cell migration, and oxidative stress. These communications differentiate MSCs into replacement cell types and modulate immune cell responses.

Despite various pieces of preclinical research evidence concerning the mechanisms of MSCs against ICH, there is still a need for further investigation about its therapeutic and preventive role to promote its use as a clinical therapy (Figure 2) since not all the promising MSC techniques have been proved clinically beneficial in a randomized controlled trial (RCT) (Fernandez Vallone et al., 2013; Azad et al., 2016; Squillaro et al., 2016; Levy et al., 2020).

## Clinical Trials and Limitations of Mesenchymal Stem Cell-Based Therapies in Clinical Translation for Intracerebral Hemorrhage

Multiple or ongoing trials have been performed that use MSCs. To date, clinical trials of MSC-based therapies for stroke first have focused on assessing safety and efficacy. A significant concern is the risk of tumor formation, and understanding the underlying biology is critical to avoid such adverse effects (Jandial and Snyder, 2009). An open-label, observer-blinded RCT of a long-term follow-up study had verified significant functional recovery after intravenous autologous MSC transplantation based on the modified Rankin Scale (mRS) score along with no difference in adverse events (Lee et al., 2010), and other clinical trials have also suggested the safety and feasibility of MSC administration in ischemic stroke (Bhasin et al., 2011, 2013). After searching the clinical trials on MSC therapy for ICH on the official website, the results display nearly six related projects (clinicaltrials.gov) (Table 1). The table shows that the clinical trials of the MSCs on ICH are rudimentary, and some of them with unknown causes have passed their completion date.

**TABLE 1:** Application of MSC-based therapy for ICH involved in clinical trials.

Trial ID no.	Phase	Cell type	Route	Status	Outcome measures	Allocation	Location
NCT03371329	1	BMSCs	IV ITV	Completed	1. Occurrence of adverse events 2. Changes in neurological function test	Non-randomized	United States
NCT02795052	Not applicable	BMSCs	IV	Recruiting	1. ADL 2. Neurologic functioning	Non-randomized	United States and United Arab Emirates
NCT04074408	2	HUMSCs	ITC	Recruiting	1. Frequency of dose limiting adverse events 2. mRS to measure the prognosis 3. NIHSS to measure stroke recovery	Randomized	China
NCT01389453	2	HUMSCs	IV	Withdrawn	1. NIHSS and FIM 2. Motor evoked potential and sensation evoked potential inspection 3. MRI	Non-randomized	China
NCT02283879	1	HUMSCs	IV	Unknown	1. Safety evaluation through vital signs, the results of clinical lab tests and adverse events 2. Improvement of infarct size measured by brain MRI 3. Modified Barthel index 4. NIHSS	Randomized	China
NCT01714167	1	BMSCs	IC	Unknown	1. Change from baseline in NIHSS at 12 months	Non-randomized	China

*IV, intravenous; ITV, intraventricular; ITC, intracavitary; IC, intracerebral; NIHSS, National Institutes of Health Stroke Scale; mRS, modified Rankin Scale; MRI, magnetic resonance imaging; FIM, function independence evaluation; ADL, activities of daily living.*

Compared with the effective positive results in preclinical research, including reducing the extent of damage, inflammation response, and free radicals, as well as reversing markers of neurodegeneration, therapeutic effects of MSC-based administration in clinical studies have shown to be unsatisfactory (Fernandez Vallone et al., 2013; Azad et al., 2016; Squillaro et al., 2016; Gutierrez-Vargas and Cardona-Gomez, 2020). Previously, the inadequate quality of preclinical tests has been recognized as a major reason for explaining the failure of translation from the preclinic research into the clinical setting (Cui L. L. et al., 2019).

Although an increasing number of investigations for early phase clinical studies of cell therapy have been conducted in stroke and other brain diseases, the convincing evidence of effectiveness is still far deficient (Kode et al., 2009; Moniche et al., 2012; Hess et al., 2017; Li J. et al., 2021). It is thought-provoking that current clinical studies of cell therapy for stroke are in the early stage of clinical trials, which fail to elucidate the critical therapeutic effects. Nevertheless, remarkable design differences between preclinical and clinical studies were detected, including cell immunogenicity, cryopreservation, cell type, recipient comorbidities, recipient sex and age, cerebral vessel occlusion modalities, the time window of cell transplantation, delivery route, and methodological limitations, which may affect clinical translation (Cui L. L. et al., 2019; Dabrowski et al., 2019). Future research should focus on applying proper biomarkers so that research investigators can estimate or discover biological targets to optimize efficacy during clinical trials. For the progress of preclinical to clinical translation, an iterative process between the clinic and the laboratory is necessary to improve the ways for MSC-based treatment and ultimately accomplish the expected results.

## Conclusion

Remarkable development has been made in understanding the underlying mechanisms of ICH-induced brain damage during the past two decades (Balami and Buchan, 2012; Fang et al., 2013; Chen S. et al., 2015; Duan et al., 2016; Bobinger and Burkardt, 2018). Most significantly, various studies have proved that MSCs can secrete various neurotrophic, angiogenic, and immunomodulatory factors displaying potential functions in the injured brain (Chen et al., 2008; Bedini et al., 2018; Galipeau and Sensebe, 2018). The neuroprotection of MSCs has been wholly verified in many studies. Improving the therapeutic effects and immunomodulatory properties of MSC application could give investigators the potential targets in the following research work.

It has been confirmed that numerous signaling molecular pathways are related to secondary brain damage with inflammatory responses (Zhou et al., 2014; Zhu et al., 2019). Although some promising results of modulation of immunologic response after ICH have been revealed in some studies, it is also critical to conduct extended trials for further verification. There are still several issues that should be addressed. Although animal models have been commonly used for inquiring into the ICH mechanisms associated with etiology and pathophysiology, advanced animal models are still lacking. Since rodents are endowed with extraordinary spontaneous rehabilitation of sensorimotor deficits and limited evidence for cognitive disorders, recent commonly used animal models did not well recapitulate the synthetic etiology of spontaneous ICH in humans (Zille et al., 2022). Therefore, some researchers suggested that the multiple common risk factors within models (including hypertension and anticoagulants) are included to perfectly imitate the clinical scenario. Due to the varied therapeutic response of different species, it is critical that potential therapies be investigated in at least two species, rather than two rodents, which ameliorates the limitations of a single species and widely enhances the applicable reliability of valid mechanisms (Hemorrhagic Stroke Academia Industry Roundtable Participants, 2018). Second, the pathological mechanisms in human ICH disease cannot be completely simulated and displayed in experimental models. Due to the paucity of translational animal models in preclinical research, large animal models were used to investigate pivotal pathophysiological parameters. Using 1.5T MRI, including structural as well as perfusion and diffusion, weighted neuroimaging reflected the critical aspects of human ICH disease and can be comparatively researched in different aspects of human ICH, including hematoma expansion, white matter injury, and hematoma evacuation (Boltze et al., 2019). Third, many studies on ICH-induced brain injury only focus on single-factor intervention. Therefore, to potentially exploit agents with multiple targets or improve the multidirectional development of drug therapy, strategies need to be well researched in further work. Last but not least, the choice of sources, quantity, and quality of MSCs are critical challenges for MSC therapy and a topic of concern for future research. Several questions related to the safety, efficacy, and critical mechanisms of MSC infusion therapy because of different aspects of cell dosage, cell source or the approaches of cell transplantation, and timing prior to clinical trials still exist and need to be further verified and elucidated (Kode et al., 2009; Singh et al., 2016; Yong et al., 2018).

## Author Contributions

ND and LW: conceptualization. GY and XF: writing—original draft preparation. MM: writing—review and editing. GY: visualization. HX, SY, and LW: project administration, science and technology project of Sichuan province, National Natural Science Foundation of China, and funding acquisition. All authors have read and agreed to the published version of the manuscript.

## Conflict of Interest

The authors declare that the research was conducted in the absence of any commercial or financial relationships that could be construed as a potential conflict of interest.

## Publisher’s Note

All claims expressed in this article are solely those of the authors and do not necessarily represent those of their affiliated organizations, or those of the publisher, the editors and the reviewers. Any product that may be evaluated in this article, or claim that may be made by its manufacturer, is not guaranteed or endorsed by the publisher.
